# Preclinical Studies on Plant Based-Antacid Formulations as New Therapies for Gastro-Oesophageal Reflux Disease

**DOI:** 10.3390/ph19010173

**Published:** 2026-01-19

**Authors:** Paola De Cicco, Nunzio Antonio Cacciola, Rebecca Amico, Barbara Romano, Umberto Di Maio, Natasa Milic, Antonino Bagnulo, Maria Francesca Nanì, Laura Viscovo, Marcello Scivicco, Raffaele Capasso, Ester Pagano, Francesca Borrelli

**Affiliations:** 1 Department of Pharmacy, School of Medicine and Surgery, University of Naples Federico II, Via D. Montesano, 49, 80131 Naples, Italy; 2 Department of Veterinary Medicine and Animal Production, University of Naples Federico II, Via F. Delpino 1, 80137 Naples, Italy; 3 Neilos S.r.l., Via Bagnulo, 95, Piano di Sorrento, 80063 Naples, Italy; 4 Department of Pharmacy, Faculty of Medicine, University of Novi Sad, Hajduk Veljkova 3, 21000 Novi Sad, Serbia; 5 Department of Agricultural Sciences, University of Naples Federico II, Via Università, 100, 80055 Portici, Italy

**Keywords:** gastro-oesophageal reflux disease, antacids, medicinal plants, gastroprotection, pyloric ligation model, gastric ulcers, myeloperoxidase (MPO) activity, gastric emptying, cytoprotective effects

## Abstract

**Background/Objectives:** Gastro-oesophageal reflux disease (GERD) refers to a disease in which stomach acid rises into the oesophagus. Currently, proton pump inhibitors (PPIs) are the most commonly used medications to treat GERD. However, long-term use of PPIs is not free from side effects, and new treatment strategies are needed. The present study was conducted to evaluate the gastroprotective potential of four different formulations containing both antiacids and medicinal plants considered useful for the treatment of GERD. **Methods:** The protective effects of the formulations on gastric ulcers in pyloric ligation-induced gastric mucosal lesions in mice were evaluated by measuring gastric emptying, the ulcer index, gastric content, total acidity, and the pH of the gastric fluid. Gastric damage was also assessed by measuring myeloperoxidase (MPO) activity. **Results:** Formulations containing *Glycyrrhiza glabra* L. or *Glycyrrhiza glabra* L. plus *Opuntia ficus-indica* Mill. and *Olea europaea* L. (formulations 3 and 4, respectively) increased gastric emptying. All formulations decreased gastro-oesophageal damage (ulceration and MPO activity) and gastric contents and had no effects on total acidity or gastric fluid pH in the pyloric ligation ulcer model. **Conclusions:** Our results show that all formulations are able to exert cytoprotective and anti-ulcerative effects. However, among the formulations, formulation 4 seems to be the most promising because of its better effects on gastric injury and gastric emptying. These results support the hypothesis of the possible use of medicinal plants in combination with antacid agents in the treatment of GERD.

## 1. Introduction

Gastro-oesophageal reflux disease (GERD) is a chronic gastrointestinal disorder characterised by the reflux of gastric contents into the oesophagus. To date, the causes of GERD are unknown, although several risk factors for the development of this disease have been identified. These risk factors include obesity, the consumption of large amounts of alcohol, coffee and fatty foods, and the use of non-steroidal anti-inflammatory drugs (NSAIDs)/aspirin [1,2]. Typical GERD symptoms include belching and heartburn. In addition, persistent acid reflux can lead to oesophageal mucosal damage and other complications, such as Barrett’s oesophagus and adenocarcinoma of the oesophagus [3]. GERD significantly affects patients’ quality of life and causes extra-oesophageal symptoms such as chest pain, chronic cough, laryngitis, insomnia, anxiety, and an unsatisfactory sex life [4]. Epidemiological studies have shown that the estimated prevalence of GERD worldwide is around 14%, with rates increasing and consequently placing a greater burden on the healthcare system [5,6]. The most commonly used treatments for GERD include symptom-relieving medications such as antacids, histamine-2 blockers, and proton pump inhibitors (PPIs) [3]. PPIs are the most effective acid inhibitors and are used as first-line treatments in patients with moderate to severe erosive oesophagitis [3]. However, long-term use of PPIs has been associated with serious adverse events, including *Clostridium difficile* infections, bacterial gastroenteritis, small intestinal bacterial overgrowth, pneumonia, chronic kidney disease, bone fractures, dementia, and myocardial infarction [7]. For these reasons, novel treatments remain desirable to avoid drug side effects.

Since the beginning of human history, herbal medicines have been rich sources of metabolites with a variety of pharmacological effects and have been used to treat various diseases, including stomach disorders. Many herbal medicines, including *Mentha x piperita* (peppermint oil), *Glycyrrhiza glabra* (liquorice root), *Melissa officinalis* (lemon balm leaves), and *Carum carvi* (caraway fruit), have been shown to relieve the symptoms of acid reflux [8]. However, the effects of these natural products are very weak, and the use of high doses leads to side effects. Currently, the combination of drugs has attracted much attention. Combinations of two or more drugs with different targets can lead to multitarget synergy and overcome the toxicity and other side effects associated with high doses of single drugs [9]. Notably, combinations of numerous extracts have reached the market and are currently used as drugs or as adjuvant therapies in the treatment of diseases [10,11].

In this work, through a literature search, we have tried to identify medicinal plants that can exert beneficial effects for the treatment of acid reflux in order to obtain formulations for their possible use in the treatment of GERD. The effects of these formulations combining antacids with plant extracts and/or derivatives have been studied in vivo on key gastrointestinal parameters such as gastric emptying (using phenol red), reflux oesophagitis, gastric secretion (volume, pH and total acidity) and gastric ulcer formation (using the pyloric ligation method). The rationale behind this combination strategy was to develop dual action formulations that provide both immediate symptomatic relief through alkaline buffering (via carbonates) and sustained therapeutic benefit through plant bioactives with muco-protective, anti-inflammatory and cytoprotective properties. The effects of the formulations were compared with those of famotidine, an H_2_ receptor antagonist that is a widely used reference drug with well-established efficacy in the pylorus ligation model [12].

## 2. Results

### 2.1. Data from Literature

The literature search identified several medicinal plants that are used to treat GERD. Based on these findings, we selected and used some medicinal plants and their derivatives to obtain four formulations. Table 1 summarises the mechanisms of action of the selected medicinal plants relevant to GERD. The selected medicinal plants and their derivatives used in the formulations are described in more detail below, thus, to emphasise the rational design of each multi-target preparation.

- *Opuntia ficus-indica* cladodes

*Opuntia ficus-indica* (L.) Mill., commonly known as prickly pear, is an angiosperm plant that belongs to the Cactaceae family. It has been reported that the cladodes of the plant exert gastroprotective effects by reducing ulcerative lesions owing to the presence of polysaccharides that appear to exert mucoadhesive effects [13]. Moreover, a very recent paper has reported that a combination of carbonates and *Opuntia ficus-indica* extract is able to protect oesophageal cells against simulated acidic and non-acidic reflux in vitro [14].

The gastroprotective effects of this plant have been confirmed by two randomised, double-blind, placebo-controlled trials, which revealed that a formulation containing sodium alginate/bicarbonate in combination with extracts of *Opuntia ficus-indica* and *Olea europaea* combined with polyphenols reduced the frequency and intensity of gastroesophageal reflux symptoms and improved the quality of life of GERD patients [15,16].

- *Olea europaea* L.

The leaves of *Olea europaea* L. (olive), a plant belonging to the Oleaceae family, have long been used in traditional medicine and have numerous pharmacological effects. Olive leaves contain compounds such as polyphenols (3-hydroxytyrosol, oleuropein and oleacein), which are able to exert antioxidant and anti-inflammatory effects, thus preventing the formation of experimentally induced gastric lesions [17,18]. In addition, olive leaves have been reported to reduce carbachol-stimulated volume, free and total acidity in rabbits and also appear to be effective in the treatment of gastro-oesophageal reflux due to their antioxidant and anti-inflammatory effects [17,19]. A clinical trial reported that a formulation containing *Olea europaea* leaves (see above) was effective in the treatment of GERD [15,16].

- *Glycyrrhiza glabra* L.

*Glycyrrhiza glabra* L. (liquorice) is a perennial medicinal plant of the Fabaceae family, widely used in traditional medicine (including Ayurveda) and extensively employed worldwide as an ingredient in tobacco products, cosmetics, foods, and pharmaceuticals [20]. Preclinical studies have shown that liquorice exerts gastroprotective effects by increasing the concentration of prostaglandins, promoting gastric mucus secretion, and prolonging the life of surface cells in the stomach through the inhibition of two enzymes, namely, 15-hydroxyprostaglandin and delta-13-prostaglandin reductase [21]. In addition, liquorice exerts anti-pepsin effects, inhibits gastrin secretion, and has antioxidant and anti-inflammatory effects. The bioactive compounds responsible for these effects have been identified as glycyrrhetinic acid, glabrene, glabridin, liquiritoside, and licochalcones A-C. Numerous clinical studies have shown that liquorice is effective or more effective than conventional medications (cimetidine, ranitidine, or antacids) in treating gastric and duodenal ulcers by reducing the ulcer size and promoting complete healing [22].

- *Mentha x piperita* L.

*Mentha x piperita* L. (mint) is a plant of the Lamiaceae family used in the symptomatic treatment of digestive disorders such as dyspepsia and gastritis. The substance responsible for its biological action is menthol. Menthol has been shown to have a protective effect on ethanol- or indomethacin-induced gastric mucosal damage by increasing mucus and PGE2 production, stimulating K^+^ATP channels, decreasing the H^+^ concentration in gastric juice, and exerting antioxidant and anti-inflammatory effects [23,24]. Moreover, oral pretreatment with menthol reduces total acid production without altering its volume, whereas intraduodenal administration of menthol decreases the gastric juice volume without lowering the H^+^ concentration in rats subjected to pylorus ligation.

- *Actinidia chinensis* Planch

*Actinidia chinensis* Planch. The golden kiwifruit is a woody perennial dioecious plant of the Actinidiaceae family. The fruit is rich in vitamin C, phenolic compounds and carotenoids with antioxidant and anti-inflammatory properties and contains a protease enzyme, actinidin. A recent preclinical study revealed that *Actinidia chinensis* has gastroprotective potential against indomethacin-induced gastric ulcers by exerting antioxidant and anti-inflammatory effects [25]. In addition, fermented golden kiwi has been reported to exert a protective effect on the gastric mucosa by reducing HCl/EtOH-induced gastric injury and reducing the gastric fluid volume, free acidity, total acidity, and pepsin activity in a pyloric ligation model [26]. However, no clinical trial has investigated the effect of golden kiwifruit on gastric injury. In contrast, a randomised crossover pilot study revealed that the fruit had no effect on gastric emptying [27].

### 2.2. Plant Extracts Characterisation

Analytical characterisation was performed on the five plant extracts (*Actinidia chinensis*, *Glycyrrhiza glabra*, *Opuntia ficus-indica*, *Menta x piperita* and *Olea europaea*) to assess their phytochemical profiles and standardisation parameters. Each extract was analysed using complementary chromatographic and spectrometric techniques [High-Performance Liquid Chromatography (HPLC), Liquid Chromatography–Mass Spectrometry (LC–MS), Thin-Layer Chromatography (TLC), Gas Chromatography with downstream Flame Ionization Detector (GC-FID) and colorimetric assays] to identify and quantify key bioactive compounds, ensuring consistency and quality of the formulations.

- *Actinidia chinensis* (Kiwi) extract

HPLC and LC–MS analyses revealed actinidin as the predominant proteolytic enzyme in the extract. The enzymatic activity was estimated at approximately 16,000 GDU per gram of extract.

- *Glycyrrhiza glabra* (Liquorice) extract

HPLC analysis confirmed the presence of the principal saponins characteristic of *Glycyrrhiza glabra*. Quantitative LC–MS determination showed a glycyrrhizic acid content ≥3% *w*/*w*, consistent with pharmacopeial quality specifications (Supplementary Figure S1).

- *Opuntia ficus-indica* extract

The total polysaccharide content of *Opuntia ficus-indica* extract was determined to be 18% of the dry extract, based on the phenol–sulphuric acid assay. Extract identity and polysaccharide fingerprint were corroborated by ^1^H-NMR analysis (Supplementary Figure S1).

- *Mentha x piperita* (Peppermint) Extract

HPLC and GC–FID analyses confirmed the chemical composition of the peppermint extract and established the total essential oil content at 3% *w*/*v* (Supplementary Figure S1).

- *Olea europaea* (Olive Leaf) extract

HPLC and LC–MS analyses showed the extract contained 3.7–4.3% total polyphenols, expressed as oleuropein equivalents (Supplementary Figure S1).

### 2.3. Gastric Emptying Evaluation

**Figure 1:**
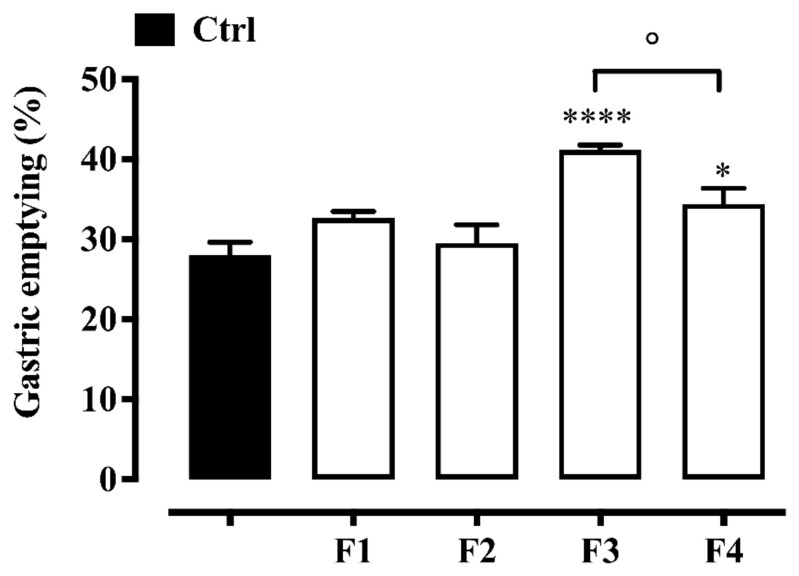
Effect of formulations 1–4 (10 µL/mouse, per oral gavage) on gastric emptying. The formulations were administered 30 min before oral administration of the marker (phenol red). Each bar represents the mean ± SEM of 9–10 mice. * *p* < 0.05 and **** *p* < 0.0001 vs. control (Ctrl, animals treated with vehicle) as assessed by one-way ANOVA followed by Tukey’s Multiple Comparison Test. ° *p* < 0.05.

Figure 1 shows the effects of formulations 1–4 at a dose of 10 µL/mouse on gastric emptying. Formulations 3 and 4, given by oral gavage, caused a significant increase in the gastric emptying rate, with formulation 3 being significantly more active than formulation 4 (Figure 1). In contrast, formulations 1 and 2 had no significant effect on gastric motility at the same dosage.

### 2.4. GERD and Gastric Ulcers Induced by Pyloric Ligation

#### 2.4.1. Macroscopic Evaluation

**Figure 2:**
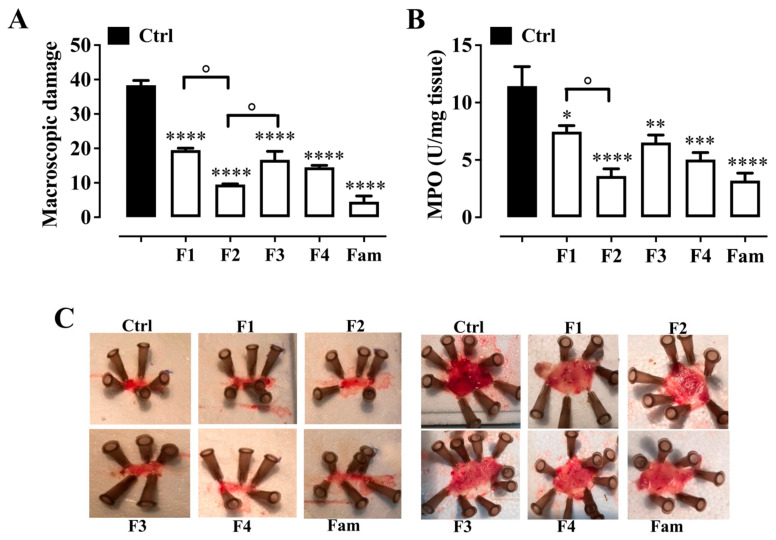
Effect of formulations 1–4 (10 µL/mouse, per oral gavage) and famotidine (Fam, 40 mg/kg, intraperitoneally, used as reference drug) on (**A**) oesophageal and gastric mucosal damage and (**B**) myeloperoxidase (MPO) activity induced by pyloric ligation in mice. (**C**) Representative image of oesophagus (**left**) and stomach (**right**) from pylorus-ligated mouse. Each bar represents the mean ± SEM of 8–9 mice (macroscopic damage) or 6 mouse tissues (MPO). * *p* < 0.05, ** *p* < 0.01, *** *p* < 0.001 and **** *p* < 0.0001 versus control (Ctrl, animals subjected to pyloric ligation and administered vehicle only), as assessed by one-way ANOVA followed by Tukey’s Multiple Comparison Test. ° *p* < 0.05.

We then examined the effects of formulations 1–4 on oesophageal and gastric mucosa damage in mice subjected to pyloric ligation. Pyloric ligation resulted in oesophageal and gastric mucosal injury characterised by an increase in blood flow (hyperaemia) and punctiform/linear ulcers (Figure 2A,C). The mucosal lesions were significantly attenuated in mice orally administered with formulations 1–4 at a dose of 10 µL/mouse (Figure 2A). Formulation 2, formulation 4 and famotidine (40 mg/kg, i.p., used as a reference drug) were the most active.

#### 2.4.2. Myeloperoxidase (MPO) Activity

To further demonstrate the efficacy of the formulations studied in reducing gastro-oesophageal mucosal damage, we examined the activity of MPO. Ligation of the pylorus resulted in a significant increase in MPO activity. Pretreatment of the mice with all the tested formulations at a dose of 10 µL/mouse significantly reduced the increased MPO activity induced by pyloric ligation (Figure 2B). Formulation 2, formulation 4, and famotidine (40 mg/kg, i.p., used as a reference drug) were the most active.

### 2.5. Gastric Volume Assessment

**Figure 3:**
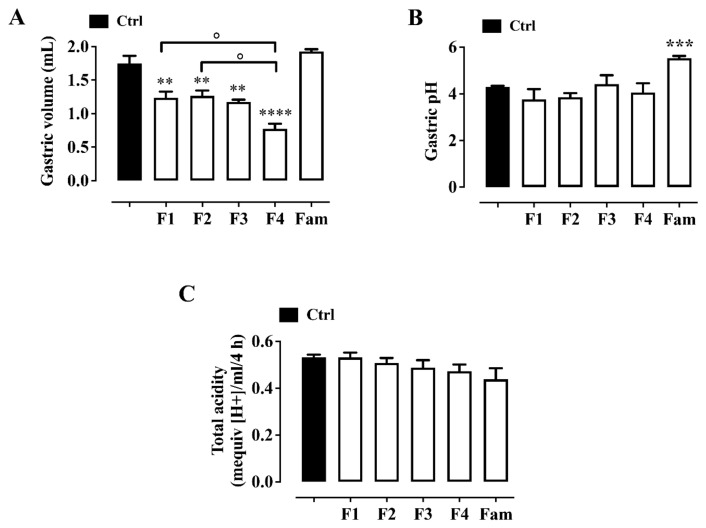
Effect of formulations 1–4 (10 µL/mouse, per oral gavage) and famotidine (Fam, 40 mg/kg, intraperitoneally, used as a reference drug) on (**A**) gastric volume, (**B**) gastric pH and (**C**) total acidity in mice subjected to pyloric ligation. The formulations were administered immediately after pyloric ligation. Each bar represents the mean ± SEM of 6–7 mice. ** *p* < 0.01, *** *p* < 0.001 and **** *p* < 0.0001 versus control (Ctrl, animals subjected to pyloric ligation and administered vehicle only) as assessed by one-way ANOVA followed by Tukey’s Multiple Comparison Test. ° *p* < 0.05.

All the tested formulations were administered orally at a dose of 10 µL/mouse, which significantly decreased the gastric volume (Figure 3A). No significant change was observed after the administration of famotidine (40 mg/kg, i.p.) (Figure 3A).

- Evaluation of gastric pH

None of the tested formulations administered at a dose of 10 µL/mouse caused significant changes in the gastric pH. An increase in gastric pH was observed in the stomachs of mice treated intraperitoneally with famotidine (Figure 3B).

- Evaluation of total gastric acidity (H^+^ concentration)

Similarly to the results of the gastric pH assessment, none of the formulations tested caused a significant change in total gastric acidity (H^+^ concentration, Figure 3C) at a dose of 10 µL/mouse. Famotidine (40 mg/kg, i.p., used as a reference drug) had no effect on total gastric acidity (Figure 3C), although a slight reduction was observed.

## 3. Discussion

Over the last ten years, the combination of medicinal plants with conventional medicine has proven to be more effective than the use of a single drug alone. The novelty of the present study lies in the development and in vivo evaluation of multifunctional formulations that combine plant-derived gastroprotective agents with low-dose antacids and a raft-forming system, with the hypothesis that such an approach can provide effective mucosal protection through mechanisms partially independent of acid suppression. In this paper, we select several medicinal plants that can be used alongside antacids for the treatment of GERD based on a comprehensive literature review. Experiments conducted on mice show that these combinations are more effective than the individual antacids due to the multiple mechanisms of action of the medicinal plants.

Literature searches in databases such as PubMed are crucial in medical therapy as they provide healthcare professionals with access to up-to-date clinical trials, systematic reviews and scientific articles. This process helps to gather reliable scientific evidence that supports therapeutic decisions based on solid data. Using PubMed, we have identified and selected several medicinal plants that are known to relieve and/or attenuate GERD symptoms or provide more effective treatment of GERD due to their multiple mechanisms of action.

Plants such as *Actinidia chinensis* (kiwi), *Glycyrrhiza glabra* (liquorice), *Mentha x piperita* (peppermint), *Opuntia ficus-indica* (prickly pear) and *Olea europaea* (olive leaf) have a positive effect on GERD by reducing inflammation and oxidative stress, protecting and healing the oesophageal mucosa and improving gastric motility [14,25,28,29,30]. In addition, some of these plants help to regulate gastric acid production and improve the function of the lower oesophageal sphincter, potentially reducing reflux episodes [29].

Given the reported beneficial effects of these medicinal plants on GERD, we formulated four different preparations combining medicinal plant extracts with antacids and a raft-forming system, which were evaluated in mice as a potential treatment for GERD. In particular, we investigated the effects of these formulations on gastric emptying, reflux oesophagitis, gastric secretion (including volume, pH and total acidity) and the development of gastric ulcers in mice.

To measure gastric emptying and gastrointestinal transit, we used the phenol red model, which is widely used to evaluate the effects of synthetic and natural products [31,32,33,34]. Our results demonstrated that, among all the formulations tested, only formulations 3 and 4 were effective in accelerating gastric emptying in mice and that this effect appears to be attributed to the presence of *Glycyrrhiza glabra* root, the only extract not included in the other formulations. Based on existing literature, it can be hypothesised that isoliquiritigenin, a flavonoid isolated from the root of *Glycyrrhiza glabra*, may contribute to the observed prokinetic effect, as it has been reported to exert prokinetic activity in previous studies [35]. Interestingly, formulation 4 had a significantly weaker effect on gastric emptying than did formulation 3, which may be due to the presence of *Mentha x piperita* leaf extract in formulation 4, which is known to reduce gastric emptying by acting on transient receptor potential channels [36]. However, a possible effect of *Opuntia ficus-indica* cladodes or *Olea europaea* leaves (or their polyphenolic compounds) on reduced gastric motility cannot be excluded. Indeed, *Opuntia ficus-indica* seeds (but not the juice of the cladodes) have been reported to reduce gastric motility and olive leaves have shown a gastrointestinal spasmolytic effect in rats [37,38].

Pyloric ligation is an animal model commonly used to mimic the effects caused by GERD in humans (e.g., gastric mucosal damage). Indeed, pyloric ligation has been reported to cause gastric mucosal breakdown in addition to excessive gastric acid secretion and to disrupt the gastric mucosal barrier [26,39,40]. Using this model, we demonstrated that oral administration of all formulations tested exerted a significant gastroprotective effect that reduced the oesophageal and gastric lesions induced by pyloric ligation. The gastro-oesophageal protective effect of these formulations is due mainly to the presence of sodium alginate, calcium carbonate and sodium bicarbonate, which either neutralise gastric acid and act as antacids (calcium carbonate and sodium bicarbonate) or induce the formation of a protective film on the gastric mucosa (sodium alginate and carbonate/bicarbonate). Interestingly, the less active formulation is formulation 1 (which contains only alginate and antacids), indicating the usefulness of the extracts in the formulation. Formulations 2 and 4, which were more active in reducing macroscopic damage and MPO activity (Formulation 2) and gastric volume (Formulation 4) compared to Formulation 1, differed from Formulation 1 by the presence of *Actinidia chinensis*, whose ability to protect the gastric mucosa from lesions in HCl/EtOH-induced gastric lesions in rats has been reported in the literature [26]. Surprisingly, formulation 3, which also contains the digestive enzymes and liquorice that are reported to have a gastroprotective effect, was less active than formulation 2 and especially formulation 4. The lower activity of formulation 3 led us to hypothesise that a possible interaction between digestive enzymes and liquorice led to a reduction in the gastro-oesophageal protective effect. In contrast, the higher activity of formulation 4 is probably due to the presence of other extracts and metabolites with anti-inflammatory and antioxidant activity (*Olea europaea*, *Opuntia ficus-indica*, *Menta x piperita* and polyphenols; the biochemical and molecular pathways involved in the extracts antioxidant and anti-inflammatory effects are reported above: see Table 1) [41,42], which mask the interaction of digestive enzymes and liquorice. However, this interaction needs further investigation. The quantification of MPO in the oesophageal and gastric mucosa provides another approach to detect tissue damage caused by pyloric ligation. MPO is a marker for the regulation of neutrophil infiltration and is widely used as an indicator of tissue damage and inflammatory responses in experimental models [12,43]. We found a significant increase in MPO activity after treatment with pyloric ligation, whereas pretreatment with all formulations resulted in a decrease in MPO activity. In terms of macroscopic damage, formulation 1 proved to be less active. Notably, famotidine, used as the reference drug, reduced oesophageal and gastric lesions as well as MPO activity induced by pyloric ligation to a similar extent as Formulations 2 and 4. However, this comparison should be interpreted with caution, as famotidine was administered intraperitoneally, whereas the tested formulations were administered orally, implying differences in pharmacokinetics and bioavailability; in particular, formulation-dependent factors and gastrointestinal residence time may contribute to the efficacy observed following oral administration.

The pyloric ligation model was also used to evaluate the effects of formulations 1–4 on gastric acid secretion. Pyloric ligation has been shown to effectively increase gastric acid secretion and accumulation in the gastric mucosa [44]. In this study, the administration of formulations 1–4 significantly decreased the gastric juice volume, with formulation 4 being the most effective of all the formulations tested. Interestingly, pretreatment with formulations 1–4 had no significant effect on the gastric acid pH or total gastric acid (H^+^ concentration), probably due to the low doses of the antacids and medicinal plants. Antacids are known to have a good safety profile; however, high doses and/or chronic use can cause acid rebound [45]. Therefore, the use of low doses of antacids could prevent this adverse effect. On the basis of the above evidence, we hypothesised that the possible therapeutic effects of the tested formulations 1–4 on peptic ulcers are independent of their acid-neutralising capacity, although some extracts have been reported to affect acid production (i.e., *Olea europaea* leaves). Although GERD is inherently a chronic disease, the pyloric ligation model remains a widely accepted and validated method to study acute gastric injury, acid secretion and mucosal protection, mechanistic features central to GERD pathophysiology. Our use of this acute model serves as a first step in evaluating the efficacy and safety of formulations containing herbal extracts and antacids in vivo. We acknowledge that future studies should investigate chronic or functional models to better simulate long-term GERD and evaluate cumulative effects. Moreover, although no toxicological effects are expected for the formulations under the experimental conditions used in this study, further investigations are warranted to assess the safety of prolonged liquorice intake, particularly in hypertensive subjects.

## 4. Materials and Methods

### 4.1. Literature Search

To identify medicinal plants with potential therapeutic benefits against gastro-oesophageal reflux disease (GERD), a literature search was conducted in the PubMed/Medline database. The following search terms and Boolean operators were used to identify herbal medicines used in preclinical and clinical studies as well as in traditional ethnobotany for the treatment of GERD: (“gastroesophageal reflux” OR “acid reflux” OR “GERD” OR “reflux oesophagitis”) AND (“medicinal plants” OR “herbal medicine” OR “phytotherapy” OR “botanicals”) AND (“gastroprotective” OR “mucosal protection” OR “anti-inflammatory” OR “antioxidant” OR “anti-acid”). Specifically, we search herbal medicines reported to neutralise gastric acid to relieve acute reflux symptoms (via carbonate buffer or synergistic botanical effects); to protect mucosal and to reduce inflammation and oxidative stress of the oesophageal and gastric mucosa.

### 4.2. Drugs and Reagents

Famotidine, phenol red, and myeloperoxidase from human leukocytes were obtained from Merck Life Science S.r.l. (Milan, Italy). Potassium sorbate, sodium benzoate, natural glycerin, calcium carbonate, sodium bicarbonate, and odium alginate were obtained from Neilos S.r.l. (Naples, Italy). All solvents and chemicals, including standards for HPLC and LC-MS analyses, were purchased from Merck Millipore. All chemicals and reagents used in this study were of analytical grade.

### 4.3. Plant Material and Extract Characterisation

*Glycyrrhiza glabra* L. (liquorice) root dry extract, *Actinidia chinensis* Planch. fruit dry extract, *Opuntia ficus-indica* Mill. (prickly pear) Cladodes dry extract, *Olea europaea* L. (common olive) leaf dry extract, *Mentha x piperita* L. (peppermint) leaf dry extract, polyphenols from peppermint and prickly pear were obtained from Neilos S.r.l. Each extract was characterised by chromatographic and spectrometric methods to confirm the identity and quantify the main bioactive markers.

The Glycyrrhiza glabra extract was first subjected to Thin-Layer Chromatography (TLC) for qualitative identification of the main saponins, followed by LC–MS analysis for quantitative determination. TLC was performed on silica gel 60 F254 plates (Merck Life Science S.r.l.), using ethyl acetate–methanol–water (100:13.5:10, *v*/*v*/*v*) as the mobile phase. Spots were visualised under UV light at 254 nm and by spraying with anisaldehyde–sulphuric acid reagent. Subsequently, HPLC analysis was carried out to confirm the presence of the principal saponins characteristic of Glycyrrhiza glabra. Quantitative determination of glycyrrhizic acid was performed by LC–MS analysis, using calibration curves generated with certified reference standards. Results were expressed as percentage (*w*/*w*) of dry ex-tract.

The Actinidia chinensis extract, standardised to a drug–extract ratio (DER) of 30:1, was analysed using High-Performance Liquid Chromatography (HPLC) and Liquid Chromatography–Mass Spectrometry (LC–MS). Chromatographic separation was performed on a C18 reversed-phase column (250 × 4.6 mm, 5 μm; Waters Corporation, Milford, MA, USA) with a binary gradient of water containing 0.1% formic acid (solvent A) and acetonitrile (solvent B). The flow rate was maintained at 1.0 mL·min^−1^, and detection was carried out at 280 nm. LC–MS analyses were conducted under positive electrospray ionisation (ESI^+^) mode to confirm the identity of the main proteolytic component.

The total polysaccharide content of *Opuntia ficus-indica* extract was determined using the phenol–sulphuric acid assay, according to standard procedures. Results were expressed as percentage (*w*/*w*) of dry extract, using glucose as a calibration standard. To corroborate the extract identity and carbohydrate fingerprint, ^1^H-NMR analysis was performed. NMR spectra were acquired and compared with reference fingerprints to confirm the presence and characteristic profile of polysaccharide components.

The *Mentha x piperita* leaf dry extract, standardised to a DER of 4:1, was characterised by HPLC and gas chromatography equipped with a flame ionisation detector (GC-FID, (Agilent Technologies, Santa Clara, CA, USA). HPLC was achieved on a C18 reversed-phase column (250 × 4.6 mm, 5 μm) using a mobile phase consisting of water (0.1% formic acid, solvent A) and acetonitrile (solvent B) under a gradient elution (10–60% B, 30 min). Detection was performed at 280 nm. GC-FID analysis was performed on the essential oil obtained by hydrodistillation using an HP-5MS column (30 m × 0.25 mm, 0.25 μm, Agilent Technologies), with oven temperature from 50 °C to 300 °C at 3 °C/min, helium as carrier gas (1 mL/min), and split injection.

The Olea europaea leaf extract was analysed by HPLC and LC–MS to determine its total polyphenol content. Chromatographic separation was achieved on a C18 reversed-phase column (250 × 4.6 mm, 5 μm) using a water–acetonitrile gradient containing 0.1% formic acid as the mobile phase. Detection was set at 280 nm. LC–MS was employed to confirm the characteristic polyphenolic profile and to assess extract purity.

### 4.4. Formulation Composition and Preparation

The compositions of the four formulations are shown in Table 2. All formulations contain mint flavouring, potassium sorbate, sodium benzoate, and natural glycerin (the latter used to increase bioavailability) as excipients. In addition, all formulations contained acid inhibitors such as calcium carbonate and sodium bicarbonate, together with sodium alginate, which provides a gastroprotective, raft-forming effect. The antacids included in the formulations were selected on the basis of their proven efficacy and safety profiles, which were supported by both clinical use and preclinical evidence. Preference was given to agents that exhibit rapid acid neutralisation, good tolerability and compatibility with plant extracts. As the formulations were developed for potential long-term use in humans, low doses of antacids and plant extracts were intentionally chosen to reduce the likelihood of acid rebound and to maintain a favourable safety profile over longer treatment periods. The formulations were prepared by diluting the components (antiacids, extracts, and excipients) in water to obtain a solution with a concentration of 10 mL of glycerin solution (Table 2). The vehicle used to suspend the formulations [10 μL glycerin (without substances/extracts) in 90 µL carboxymethylcellulose (CMC)/mouse; final volume 100 µL] did not significantly affect the responses under study.

### 4.5. Animals

Male CD1 mice (6–8 weeks old, weighing 20–25 g) were purchased from Charles River Lab. (Sant’Angelo Lodigiano, Italy). The mice were housed in polycarbonate cages under controlled conditions (temperature 23 ± 2 °C, humidity 60 ± 2% and a day–night cycle of 12 h). The animals had free access to water and food (standard rodent chow purchased from “Mucedola Mangimi”, Settimio Milanese, Italy), except for food deprivation, 18 h before gastric emptying analysis and 24 h before pyloric ligation. During the preoperative fasting period, water was allowed ad libitum. After pyloric ligation, however, animals were maintained under complete fasting conditions, with no access to food and water, to avoid interference with the assessment of gastric parameters, including gastric volume, pH, total acidity, and acid secretion. In the pyloric ligation study, a normal blank control group (i.e., non-GERD-induced animals) was not included in this study.

### 4.6. Gastric Emptying

Gastric emptying was studied via the method described by [46]. The mice randomly assigned to our five experimental groups (ctrl, F1–F4,) received 0.2 mL of a 0.05% phenol red solution (in 1.5% CMC by oral gavage (control mice received vehicle only). The mice were sacrificed immediately (0 min) or 20 min after phenol red administration. The abdomen was then opened, and the stomach was isolated and mixed with 4 mL of saline for 20 s. For phenol red measurement, the stomachs were treated with 2 mL of 1 M NaOH solution and mixed thoroughly for 20 s. The phenol red intensity was measured via a spectrophotometer (560 nm). Gastric emptying was evaluated according to the following formula: Gastric emptying (%) = 100 × [1 − (phenol red amount after 20 min/phenol red amount at time 0 min)].

### 4.7. Gastro-Oesophageal Reflux and Peptic Ulceration

Gastro-oesophageal reflux and peptic ulcer models were studied via the pyloric ligation method [47]. Briefly, all animals that had fasted for 24 h were randomly assigned to six experimental groups (ctrl, F1–F4 and famotidine) and anaesthetised with ketamine (50 mg/kg) and xylazine (5 mg/kg), and the abdomen was opened through a longitudinal incision of approximately 2 cm. The stomach was localised, and the pyloric sphincter was ligated with a suture. After 4 h (time required to detect a submaximal lesion of the oesophageal mucosa) [48], the mice were sacrificed in a CO_2_-saturated atmosphere, and the oesophagus and stomach were harvested to assess macroscopic oesophageal and gastric mucosal damage, the degree of oesophageal and gastric inflammation (myeloperoxidase activity), and gastro-oesophageal reflux parameters (pH, gastric content, and total acidity).

#### 4.7.1. Microscopic Mucosal Damage

Epithelial damage was assessed by microscopic evaluation of the oesophageal and gastric mucosa. For this purpose, the oesophagus (along the long axis) and stomach (along the greater curvature) were opened, fixed on a polystyrene support, and viewed with a dissecting microscope with square grids (×10). Epithelial damage was quantified using a score that considered the severity and extent of mucosal erosion and hyperaemia. Damage was summed per tissue and expressed as macroscopic damage. All investigators who measured the lesions were blinded to the treatments given to the animals.

#### 4.7.2. Mucosal Inflammation (MPO Activity)

Oesophageal and gastric inflammation was assessed by measuring myeloperoxidase (MPO) activity. MPO is an enzyme stored in the azurophilic granules of polymorphonuclear neutrophils and is widely used as a marker of neutrophil infiltration caused by an inflammatory stimulus [49]. Approximately 50 mg of tissue was homogenised in 1 mL of lysis buffer [0.5% hexadecyltrimethylammonium bromide in 10 mM 3-(N-morpholino) propanesulfonic acid]. The homogenates were centrifuged at 15,000× *g* for 20 min at 4 °C, and an aliquot of the supernatant was incubated with sodium phosphate buffer (NaPP pH 5.5) and 16 mM tetramethylbenzidine. After 5 min of incubation at room temperature, hydrogen peroxide (H_2_O_2_; 9.8 M in NaPP) was added, and the reaction was stopped with acetic acid (2 M). The resulting blue dye concentration was measured via a spectrophotometer (650 nm). The absorbance values were interpolated with a standard curve of recombinant MPO, and the results are expressed as MPO units/mg tissue.

### 4.8. Gastro-Oesophageal Reflux Parameters

The gastric contents from -mice subjected to pyloric ligation were collected and centrifuged at 5000 rpm for 15 min at 4 °C. The volume of each sample was measured, and then the samples were diluted with deionised water to a volume of 2 mL. The acid concentration was measured by chemical titration with 2% phenolphthalein and 0.01 N NaOH. The values are expressed as the mean H+/mL/4 h.

### 4.9. Pharmacological Treatment

The formulations were administered by oral gavage 30 min before oral administration of the marker for measuring gastric emptying (phenol red) and immediately after pyloric ligation. The formulations were suspended in 0.5% CMC to administer a final volume of 100 µL of solution per mouse [10 µL glycerine solution (which contains the substances and extract) in 90 µL of CMC; see Table 1 to know the amount of each substance/extract contained in 10 µL glycerine solution]. Famotidine, administered intraperitoneally after pyloric ligation at a dose of 40 mg/kg, served as the reference drug. In each set of experiments, all animals received the corresponding vehicle for any treatment not given.

### 4.10. Statistical Analysis

Statistical analysis (using GraphPad Prism 10.3.1) was performed using one-way ANOVA followed by Tukey’s multiple comparison test to compare multiple groups. Differences were considered statistically significant at *p* < 0.05. The data are expressed as the mean ± mean standard error (SEM) of n experiments.

## 5. Conclusions

In conclusion, the present study demonstrates that formulations combining antacid agents with selected medicinal plant extracts exert significant gastroprotective and anti-ulcerative effects in a preclinical model of gastro-oesophageal reflux–related gastric injury. All tested formulations were able to reduce oesophageal and gastric mucosal damage, inflammatory infiltration, and gastric content volume, without significantly altering gastric pH or total acidity, suggesting a cytoprotective mechanism largely independent of acid neutralisation. Among the tested preparations, formulation 4 emerged as the most promising, showing superior efficacy in reducing gastric injury, inflammatory markers, and gastric volume, together with a favourable effect on gastric emptying. These effects are likely attributable to the complementary and multitarget actions of its botanical components, including mucoadhesive, antioxidant, anti-inflammatory, and motility-modulating properties, combined with the protective raft-forming activity of sodium alginate and the buffering action of low-dose antacids.

Overall, these findings support the concept that plant-based antacid formulations may represent a valuable alternative or adjunct to conventional pharmacological therapies for GERD, potentially reducing reliance on long-term proton pump inhibitor treatment and its associated adverse effects. Importantly, the investigated formulations may also serve as prototypes for the development of future pharmaceutical products that integrate antacid activity with plant-derived gastroprotective mechanisms. Although the pyloric ligation model reflects an acute condition, it provides relevant mechanistic insights into mucosal protection and gastric injury. Further studies using chronic and functional models, as well as clinical investigations, are warranted to confirm efficacy, long-term safety, and translational relevance, particularly with respect to prolonged use and specific patient populations.

## Figures and Tables

**Table 1:** Summary of the relevant mechanisms of action of the medicinal plants used in the four formulations for GERD treatment.

Medicinal Plant	Mechanisms Relevant to GERD	References
*Opuntia ficus-indica* L.	Mucoadhesive effect; barrier formation; reduction in ulcerative lesions; oesophageal cell protection	[13,14,15,16]
*Olea europaea* L.	Antioxidant (by enhancing antioxidant defences) and anti-inflammatory (by inhibiting the NF-κB and COX-2 pathways) activities; acid secretion inhibition; mucosal protection	[15,16,17,18,19]
*Glycyrrhiza glabra* L.	Anti-inflammatory (due to inhibition of NF-κB) effect. Increased prostaglandins; mucus secretion; anti-pepsin and anti-gastrin effects; mucosal healing	[20,21,22]
*Mentha x piperita* L.	Increased mucus and PGE_2_; stimulation of K^+^ATP channels; acid secretion reduction; anti-inflammatory and antioxidant activity	[23,24]
*Actinidia chinensis* Planch	Antioxidant and anti-inflammatory activity reduce pro-inflammatory markers such as TNF-α, IL-1β, and IL-6, and also activates antioxidant pathways via Nrf2; gastric protection in ulcer models; reduction in acid and pepsin secretion	[25,26,27]

ATP, Adenosine triphosphate; COX-2, Cyclooxygenase 2; IL-1β, Interleukin-1 beta; IL-6, Interleukin-6; NF-κB, nuclear factor kappa-light-chain-enhancer of activated B cells; Nrf2, Nuclear factor erythroid 2-related factor 2; PGE_2_, Prostaglandin E2; TNF-α, Tumour necrosis factor-alpha.

**Table 2:** Composition of the four tested formulations (for 10 mL), highlighting both the antacid components and the botanical or functional additives.

Component	Formulation 1	Formulation 2	Formulation 3	Formulation 4
Sodium alginate (500 mg)				
Calcium carbonate (266.66 mg)	✓	✓	✓	✓
Sodium bicarbonate (150 mg)				
Mint flavour (5 mg)				
Potassium sorbate + Sodium benzoate (20 mg)	✓	✓	✓	✓
*Actinidia chinensis* planch (kiwi) fruit dry extract (20 mg)		✓	✓	✓
*Glycyrrhiza glabra* L. roots dry extract (30 mg)			✓	✓
*Opuntia ficus-indica* Mill. (prickly pear) cladodes dry extract (52 mg)				✓
*Olea europaea* L. (olive) leaves dry extract (38 mg)				✓
*Mentha x piperita* L. (Mint) leaves dry extract (30 mg)				✓
Polyphenols from Olive trees and Prickly Pears (6 mg)				✓
Vegetable glycerin (up to 10 mL)	✓	✓	✓	✓

## Data Availability

The original contributions presented in this study are included in the article/Supplementary Materials. Further inquiries can be directed to the corresponding authors.

## Supplementary Materials

The following supporting information can be downloaded at https://www.mdpi.com/article/10.3390/ph19010173/s1. Figure S1: High-performance liquid chromatography (HPLC) profile of *Olea europaea* (leaves), *Glycyrrhiza glabra* L. (root) and *Mentha piperita* L. (leaves), and ^1^H-NMR fingerprint spectrum of *Opuntia ficus-indica* extract (cladodes).
