# Supplementary material for: Preclinical Studies on Plant Based-Antacid Formulations as New Therapies for Gastro-Oesophageal Reflux Disease
Source: Pharmaceuticals (Basel). 2026 Jan 19;19(1):173. doi: 10.3390/ph19010173 (PMC12844621; doi:10.3390/ph19010173)
Supplement: Supplementary file 1 [file pharmaceuticals-19-00173-s001.zip › pharmaceuticals-3923876-supplementary.pdf]

Supplementary materials

A

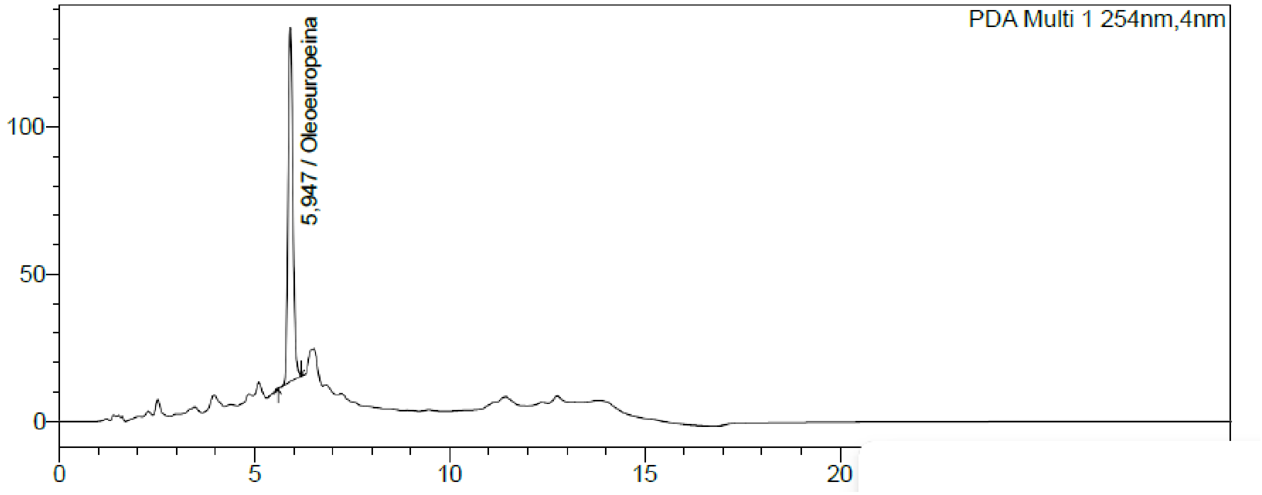

B

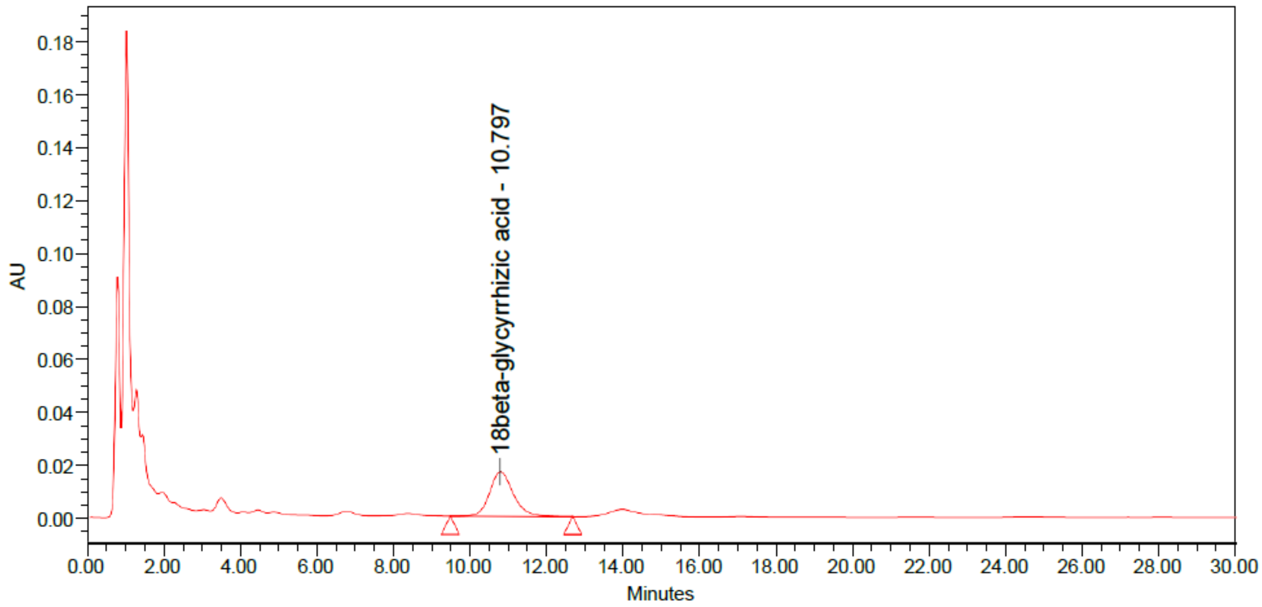

C

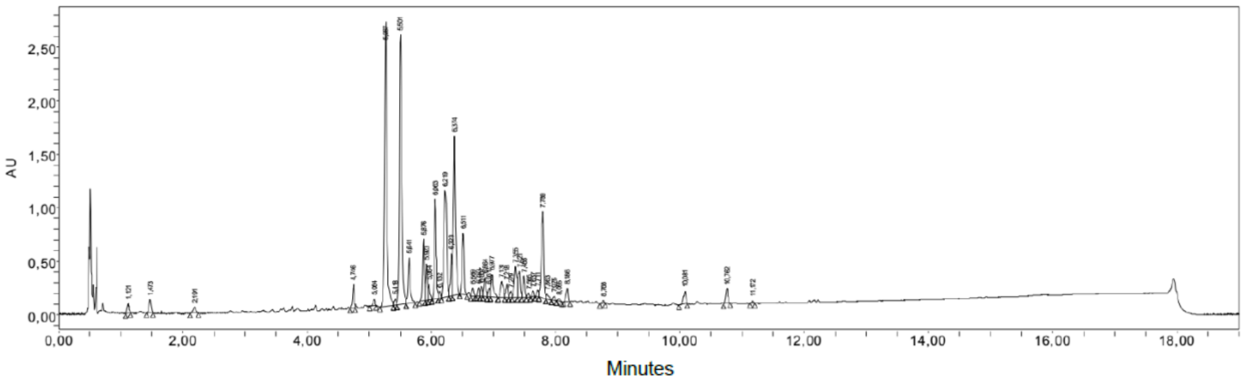

D

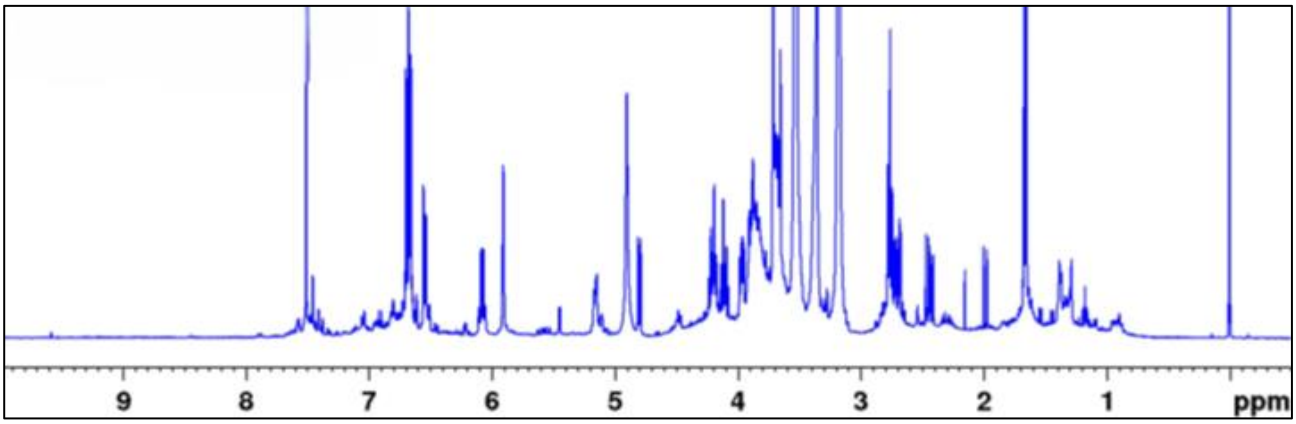

Supplementary Figure S1. High-performance liquid chromatography (HPLC) profile of the following plant extract: **A)** *Olea europaea* (leaves), **B)** *Glycyrrhiza glabra* L. (root) and **C)** *Mentha piperita* L. (leaves). **D)** ^{1}H-NMR fingerprint spectrum of *Opuntia ficus-indica* extract (cladodes).
